# Structural Basis for Apoptosis Inhibition by Epstein-Barr Virus BHRF1

**DOI:** 10.1371/journal.ppat.1001236

**Published:** 2010-12-23

**Authors:** Marc Kvansakul, Andrew H. Wei, Jamie I. Fletcher, Simon N. Willis, Lin Chen, Andrew W. Roberts, David C. S. Huang, Peter M. Colman

**Affiliations:** 1 The Walter and Eliza Hall Institute of Medical Research, Parkville, Victoria, Australia; 2 Department of Medical Biology, University of Melbourne, Parkville, Victoria, Australia; University of Southern California School of Medicine, United States of America

## Abstract

Epstein-Barr virus (EBV) is associated with human malignancies, especially those affecting the B cell compartment such as Burkitt lymphoma. The virally encoded homolog of the mammalian pro-survival protein Bcl-2, BHRF1 contributes to viral infectivity and lymphomagenesis. In addition to the pro-apoptotic BH3-only protein Bim, its key target in lymphoid cells, BHRF1 also binds a selective sub-set of pro-apoptotic proteins (Bid, Puma, Bak) expressed by host cells. A consequence of BHRF1 expression is marked resistance to a range of cytotoxic agents and in particular, we show that its expression renders a mouse model of Burkitt lymphoma untreatable. As current small organic antagonists of Bcl-2 do not target BHRF1, the structures of it in complex with Bim or Bak shown here will be useful to guide efforts to target BHRF1 in EBV-associated malignancies, which are usually associated with poor clinical outcomes.

## Author Summary

Altruistic suicide of infected host cells is a key defense mechanism to combat viral infection. To ensure their own survival and proliferation, certain viruses, including Epstein-Barr virus (EBV), have mechanisms to subvert apoptosis, including the expression of homologs of the mammalian pro-survival protein Bcl-2. EBV was first identified in association with Burkitt lymphoma and it is also linked to certain Hodgkin's lymphomas and nasopharyngeal carcinoma. Whereas increased expression of Bcl-2 promotes malignancies such as human follicular lymphoma, the precise role of the EBV encoded Bcl-2 homolog BHRF1 in EBV-associated malignancies is less well defined. BHRF1 is known to bind the pro-apoptotic BH3-only protein Bim, and here we demonstrate that it also binds other pro-apoptotic proteins (Bid, Puma, Bak) expressed by host cells. Crystal structures of BHRF1 with the BH3 regions of Bim and Bak illustrate these interactions in atomic detail. A consequence of BHRF1 expression is marked resistance to a range of cytotoxic agents, and we show that its expression renders a mouse model of Burkitt lymphoma untreatable. As current antagonists of Bcl-2 do not target BHRF1, our crystal structures will be useful to guide efforts to target BHRF1 in EBV-associated malignancies, which are usually associated with poor clinical outcomes.

## Introduction

To combat invading viruses, altruistic suicide of the infected host cells may be initiated to rapidly and efficiently eliminate the pathogen [1], [2]. Often, this response is a critical component of host defences [1], [2]. Consequently, many viruses have co-evolved adaptive mechanisms to subvert apopt osis, thereby ensuring their own survival and propagation. Some viruses, such as Epstein-Barr virus (EBV), encode homologs of the mammalian pro-survival protein Bcl-2 [3], [4], [5]. EBV was first identified in association with Burkitt lymphoma and it is also linked to other lymphoid malignancies (Hodgkin's lymphoma, post-transplant lymphoproliferative disorders) and nasopharyngeal carcinoma [6]. Whereas increased expression of Bcl-2 promotes malignancies such as human follicular lymphoma [7], the precise role of the EBV encoded Bcl-2 homolog BHRF1 in EBV-associated malignancies is less well defined.

However, more recent studies link BHRF1 to the transformation of primary B lymphocytes [8] and to lymphomagenesis [9]. Since overactivity of the oncogene *myc* is obligatory for Burkitt lymphoma [10], [11], [12], expression of BHRF1 may be necessary to block *myc*-induced apoptosis, akin to the striking synergy observed between Bcl-2 and *myc* during B cell transformation [13], [14]. Of note, the constitutive expression of BHRF1 permits lymphoblastoid immortalization by EBV and their prolonged survival [9], and together with expression of BHRF1 during normal B cells transformation [8] suggests a role for BHRF1 in post-transplant lymphoproliferative disease. Although confirmed BHRF1 expression has been shown in only a subset of Burkitt lymphomas [9], [15], it is plausible that BHRF1 plays a central role in the maintenance of this subset of Burkitt lymphomas as Bcl-2 overexpression is rare in this disease.

As BHRF1 may be central for developing and maintaining certain EBV-associated lymphomas, we investigated if BHRF1 can modulate responses to therapy in experimental models. If so, BHRF1 represents an attractive drug target since normal cells may well be spared by its selective antagonism. Here, we show that BHRF1 potently confers chemoresistance, and importantly, it adversely impacts upon survival in a mouse model of Burkitt lymphoma. BHRF1 acts by sequestering a subset of the pro-apoptotic Bcl-2 family proteins; we show here the 3D structures of it in complex with BH3 domains of two, Bim and Bak, which may provide the basis for developing small molecule inhibitors of BHRF1 to improve the generally poor prognosis in EBV-associated malignancies.

## Results/Discussion

### BHRF1 counters apoptosis induced by multiple chemotherapeutic agents

**Figure 1:**
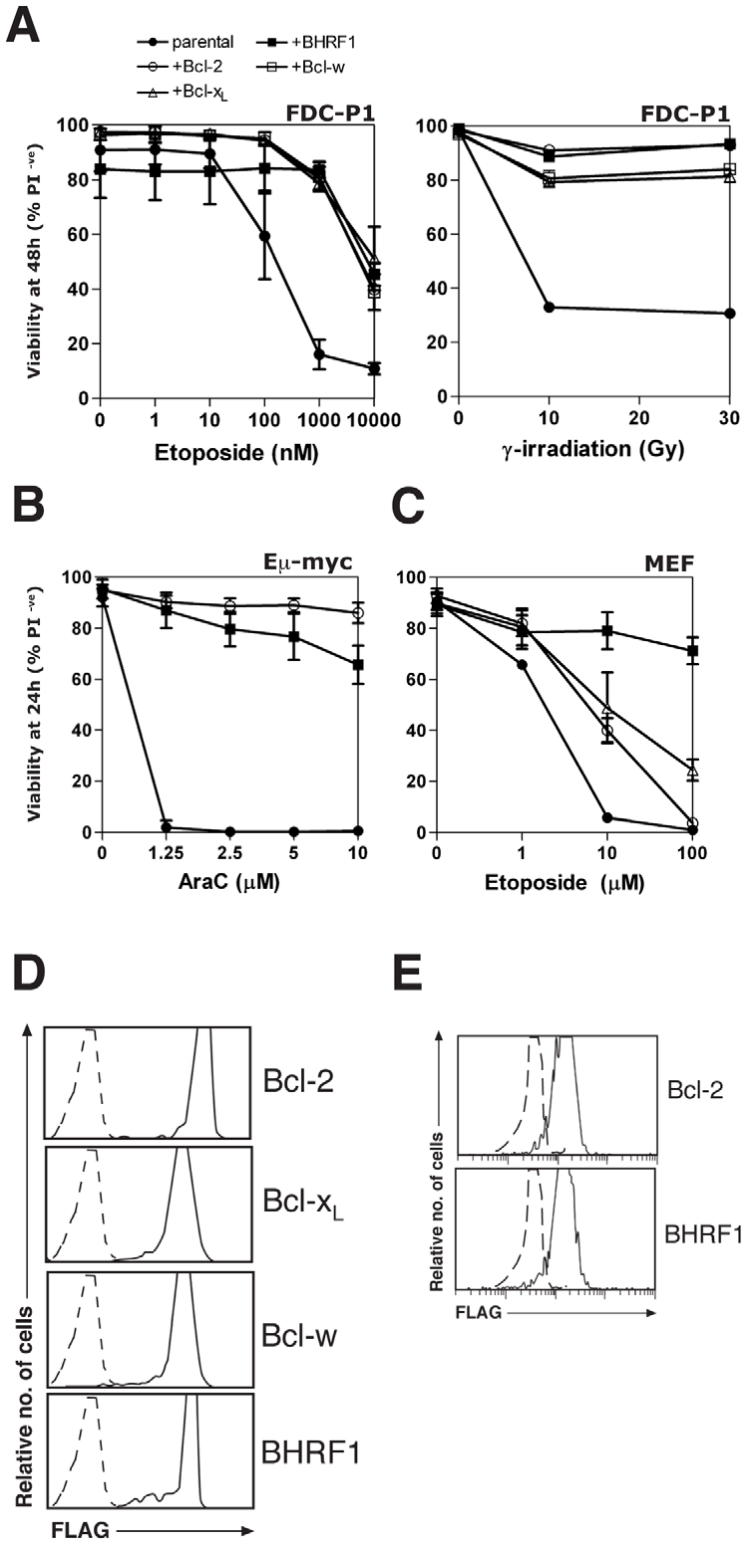
BHRF1 protects cells from diverse apoptotic stimuli. (A) Viability of FDC-P1 cells stably overexpressing BHRF1, Bcl-2, Bcl-x_L_, Bcl-w or vector, treated with 0-10 µM etoposide (left) or 0–30 Gy γ-irradiation (right) and cultured for 48 h. (B) Eµ-*myc* pre-B-cell tumor cells were stably transfected with BHRF1, Bcl-2 or vector were exposed to 0–10 µM cytosine arabinoside (Ara-C) for 24 h. (C) MEFs overexpressing BHRF1, Bcl-2, Bcl-x_L_ or vector were treated with 0–100 µM etoposide for 24 h. (D) Bcl-2, Bcl-x_L_, Bcl-w and BHRF1 are expressed at comparable levels in FDC-P1 cells. FLAG-tagged wild-type Bcl-2, Bcl-x_L_, Bcl-w and BHRF1 were stably expressed in FDC-P1 cells. Protein expression was evaluated using flow cytometry after staining fixed cells with an anti-FLAG antibody, followed by an anti-mouse FITC secondary antibody. Controls (dotted lines) indicate staining of cells expressing empty vector. (E) BHRF1 and Bcl-2 are expressed at comparable levels in Eµ-*myc* pre-B-cell tumor cells. Pre-B-cell tumor cells derived from Eµ-*myc* transgenic mice were stably transfected with FLAG-tagged BHRF1, Bcl-2 or an empty control vector. Protein expression was evaluated using flow cytometry after staining fixed cells with an anti-FLAG antibody, followed by an anti-mouse FITC secondary antibody. Controls (dotted lines) indicate staining of cells expressing empty vector. Cell viability was determined by PI exclusion; data shown are means ±1 SEM of 3 independent experiments except for the representative γ-irradiation experiment shown in (A).

Using cultured cell lines, we tested the ability of BHRF1 to confer resistance against a range of apoptotic stimuli, especially those used for cancer chemotherapy. Stable expression of BHRF1 in FDC-P1 mouse myelomonocytic cells conferred resistance to etoposide or γ-irradiation (Fig. 1A) comparable to that observed in cells expressing similar levels of Bcl-2, Bcl-x_L_ or Bcl-w (Fig. 1D). It also inhibited apoptosis induced by other cytotoxics including cytosine arabinoside (Ara-C), doxorubicin, etoposide and staurosporine in other cell lines (Figs. 1B, 1C, S1 and data not shown), comparable to that observed in cells expressing similar levels of Bcl-2 (Fig. 1E). Thus, BHRF1, like its mammalian counterparts, inhibits apoptosis induced in multiple cell types by diverse chemotherapeutic agents.

### BHRF1 preserves mitochondrial function by inhibiting the activation of Bax and Bak

**Figure 2:**
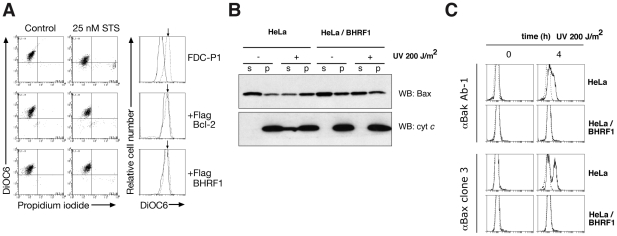
BHRF1 inhibits loss of mitochondrial transmembrane potential and Bax/Bak activation. (A) FDC-P1 cells expressing BHRF1, Bcl-2 or a control vector were treated with 25 nM staurosporine for 24 h. Outer mitochondrial transmembrane potential (Δψ_m_) was assessed by DiOC_6_(3) uptake. (B) HeLa cells treated with 200 J/m^2^ UV-irradiation were analyzed at 4 h for Bax translocation and cytochrome *c* release by immunoblotting after fractionation into soluble (s) and pellet (p) fractions. (C) Bax and Bak activation in UV-irradiated HeLa cells were assessed at 4 h using the conformation-specific mouse anti-Bak clone Ab-1 (Calbiochem) or mouse anti-Bax clone 3 antibodies [53].

To ascertain precisely how BHRF1 interferes with apoptosis signaling, we examined mitochondrial outer membrane permeabilization (MOMP) [16] in FDC-P1 cells after treatment with staurosporine. Whereas MOMP occurred rapidly in the parental cells, as indicated by the reduced uptake of the mitochondrial dye DiOC_6_(3), it was inhibited in cells expressing BHRF1 or Bcl-2 (Fig. 2A), implicating an anti-apoptotic effect upstream of mitochondrial damage. When the mediators of mitochondrial damage (Bax and Bak) were examined, we found that BHRF1 inhibited translocation of Bax from the cytosol (Fig. 2B) and activation of Bax and Bak was abrogated, as their conformational alteration associated with activation is blocked (Fig. 2C). Consistent with these observations, BHRF1 also inhibited the release of cytochrome *c* from within the mitochondria (Fig. 2B).

We conclude that BHRF1 must exert its anti-apoptotic effect at the level of, or prior to, Bax and Bak activation, consistent with a report that BHRF1 prevented Bax and Bak conformational change, oligomerization and activation of the initiator caspase, caspase-9 [17]. How then might BHRF1 inhibit activation of these essential cell death mediators?

### BHRF1 interacts with a sub-set of pro-apoptotic Bcl-2 family proteins

**Figure 3:**
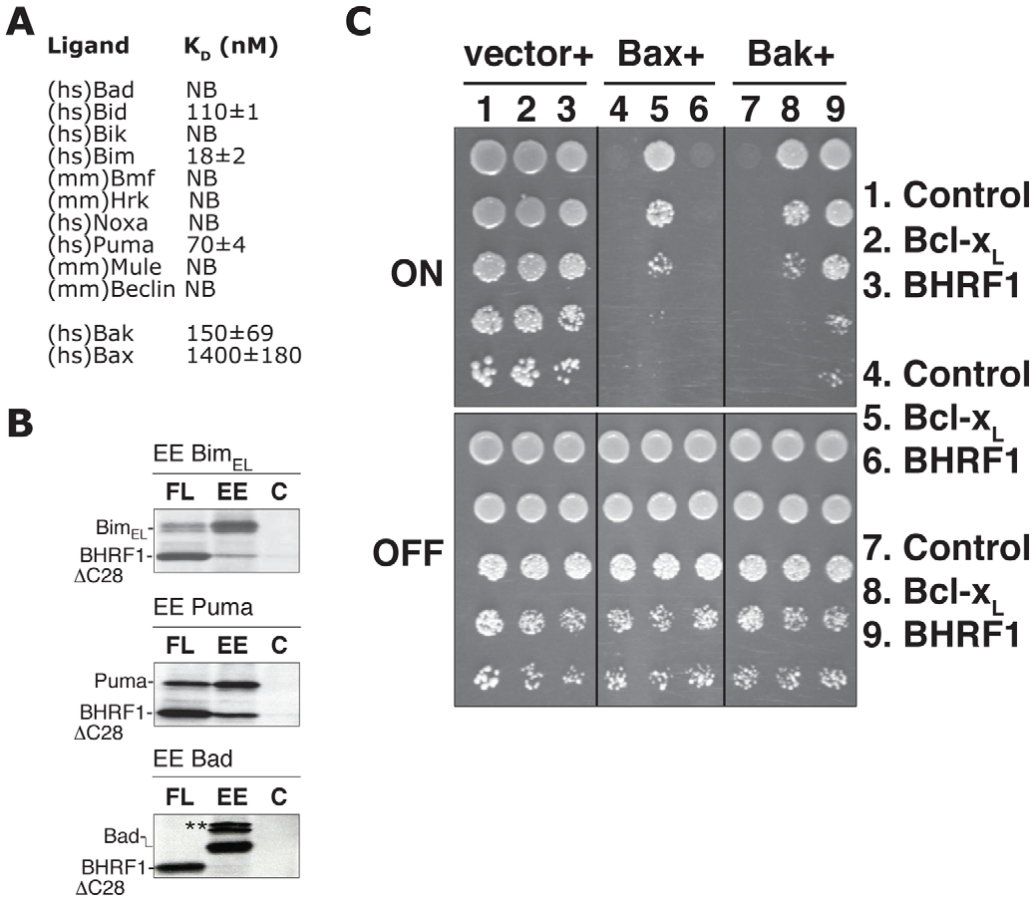
BHRF1 binds a subset of pro-apoptotic Bcl-2 family proteins to counter apoptosis. (A) The affinity of recombinant BHRF1ΔC31 for BH3 domain peptides (26-mers, except for a Bax 28-mer and a Bid 34-mer) was assessed using isothermal titration calorimetry (ITC). K_d_ values (in nM) are the means of 3 experiments ± SD. NB: no binding detected. (B) Lysates prepared from ^35^S-labeled HEK-293T cells co-expressing FLAG-tagged BHRF1 and EE-tagged BH3-only proteins were immunoprecipitated with mouse monoclonal antibodies recognizing the FLAG (FL), EE, or an irrelevant control (C) tag. **Endogenous 14-3-3 associating with Bad [56]. (C) Yeast co-transformed with constructs encoding Bax or Bak and the indicated pro-survival proteins, each under the control of an inducible (GAL) promoter, were spotted onto inducing galactose (“ON”) or repressing glucose (“OFF”) plates as 5-fold serial dilutions. Images are representative of 2 independent experiments.

It is most likely that BHRF1 acts to abort cell death initiation by sequestering the endogenous pro-apoptotic mammalian Bcl-2 family members. Thus, we assessed the ability of a recombinant C-terminally truncated form of BHRF1 to directly bind peptides spanning the BH3 domains of pro-apoptotic Bcl-2 proteins using isothermal titration calorimetry (ITC). Binding was observed with peptides from the BH3-only proteins Bim (K_D_ = 18 nM), Puma (70 nM) or Bid (110 nM) (Fig. 3A). No detectable binding was observed with peptides from other BH3-only proteins, or with Mule and Beclin-1, other proteins harboring a BH3 domain. We confirmed the interaction of intact BHRF1 in mammalian cells with selected full-length BH3-only proteins in co-immunoprecipitation assays (Fig. 3B). These results closely mirror those obtained in solution competition assays, using either fluorescence polarization [18] or surface plasmon resonance [19]. Thus, BHRF1 probably antagonizes a subset of the BH3-only proteins by direct sequestration. Interestingly, the ones targeted (Bid, Bim, Puma) are potent inducers of apoptosis, either because they neutralize most, if not all, the mammalian pro-survival proteins [20], [21] or because they can directly activate Bax or Bak [22], [23], [24].

We also investigated if BHRF1, in a manner similar to some mammalian [21], [25] and viral Bcl-2 proteins [26], [27], might directly bind Bax and Bak, the downstream mediators of mitochondrial damage. Recombinant BHRF1 bound a 26-mer Bak BH3 peptide (K_D_ = 150 nM), but only weakly (>1 µM) to Bax BH3 (Fig. 3A). Consistent with the binding data, we observed that BHRF1 could directly counter Bak (Fig. 3C), but not Bax, when these proteins were expressed in yeast [28]. This heterologous model system is suited for studying the functional interactions by circumventing potential complications due to the presence of endogenous Bcl-2 family proteins in mammalian cells and avoids the use of detergents that may artificially induce or disrupt interactions between Bcl-2 family proteins [29]. Our assay is based on the observation that overexpression of Bax or Bak in yeast is lethal, even though yeasts do not express Bcl-2 family members and do not undergo apoptosis. Nonetheless, co-expression of Bcl-2, Bcl-x_L_, Mcl-1, or A1 with Bax and Bak can suppress death induced in yeast [30], thus reconstituting key aspects of the mammalian apoptotic machinery. Our observation that BHRF1 could counter Bak (Fig. 3C), but not Bax-induced yeast death when these proteins were expressed in yeast suggests that BHRF1 is only able to directly neutralize Bak, but not Bax.

Taken together with the previous reports that BHRF1 interacts with the full-length Bak, but not with Bax [17], [31], [32], we conclude that BHRF1 can keep Bak inactive by direct binding (Fig. 3A, 3C, [19]), but must inhibit Bax indirectly, presumably by its ability to sequester BH3-only proteins such as Bim (Fig. 3A, 3B, [31]). It will therefore be interesting to investigate which pro-apoptotic protein is the critical target for BHRF1 in diverse cell types, especially those targeted by EBV during oncogenesis. It is noteworthy that in some lymphoid cells, the pro-survival action of BHRF1 tracked with its ability to bind Bim [31], which is critical for apoptosis induced by multiple stimuli in this cell type [33] and plays a role in suppressing *myc*-driven lymphomagenesis [34].

### Structural basis for the engagement of BH3 domains by BHRF1

**Figure 4:**
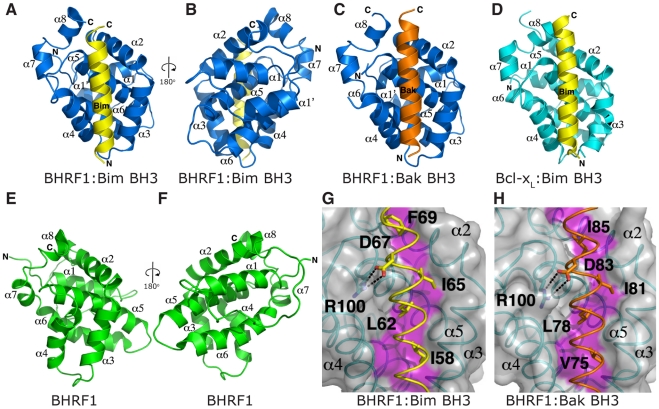
BH3 peptides bind BHRF1 in a canonical binding groove. (A–B) BHRF1 (blue) in complex with the Bim BH3 domain (yellow). BHRF1 helices are labeled α1, α1′, α2-8. The view in (A) is into the hydrophobic binding groove formed by helices α3-5, while (B) is rotated by 180° through the vertical axis to illustrate helix α1′. (C) BHRF1 (blue) in complex with the Bak BH3 domain (orange). The view is as in (A). (D) Bcl-x_L_ (cyan) in complex with the Bim BH3 domain [36]. The view is as in (A). (E–F) Unliganded BHRF1 ([35]; in green). The view in (E) is similar to (A), while (F) is comparable to (B). The binding groove is occluded by the near parallel arrangement of helices α3 and α4. (G) Detailed view of the BHRF1:Bim complex interface. The BHRF1 surface, backbone and binding groove are shown in grey, cyan and magenta respectively, while Bim BH3 is shown in yellow. The four key hydrophobic residues of Bim (I58, L62, I65 and F69; numbering based on human Bim_L_) protruding into the binding groove, as well as the conserved salt-bridge formed by Bim D67 and BHRF1 R100 are labeled. (H) Detailed view of the BHRF1:Bak complex interface. Colour scheme is as for (G) with Bak BH3 shown in orange. The four key hydrophobic residues of Bak (V75, L78, I81 and I85) protruding into the BHRF1 binding groove, as well as the conserved salt-bridge formed by Bak D83 and BHRF1 R100 are labeled.

Our data (Fig. 3) and previously published studies [18] suggest that BHRF1, like its mammalian counterparts, inhibits cell death by sequestering endogenous pro-apoptotic Bcl-2 proteins. However, the structural basis for this is unclear since a previously published structure of C-terminally truncated BHRF1 lacked the characteristic hydrophobic surface groove responsible for interaction with the BH3 domains [35]. We have therefore determined crystal structures of BHRF1 in complex with its key targets, the BH3 domains of Bim (Fig. 4A–B, Table 1) and Bak (Fig. 4C, Table 1).

**Table 1:** Crystallographic statistics.

Crystal	BHRF1:Bim BH3	BHRF1:Bim BH3	BHRF1:Bak BH3
*Data collection and phasing*	Derivative MeHg	Native	Native
Spacegroup	P3_2_21	P3_2_21	P3_2_21
Resolution range (Å)	50 - 2.7	50 – 1.5	50 - 2.05
Unique reflections	6110	33924	13668
Multiplicity a	7.2 (5.5)	9.6 (6.7)	10.0 (5.1)
Completeness (%) a	99.1 (94.3)	98.8 (93.1)	99.5 (95.8)
I/σI		40.7 (2.4)	33.9 (2.2)
R_merge_ a,b	0.088 (0.346)	0.043 (0.568)	0.064 (0.497)
R_deriv_ c	0.212		
R_cullis_ (centric/acentric) d	0.653/0.609		
Phasing power (centric/acentric) e	1.46/1.39		
*Refinement*			
Resolution range (Å)		50 – 1.5	20 - 2.05
Reflections (working set/test set)		32174/1705	12945/668
Protein atoms		1466	1437
Solvent atoms		116 H_2_O, 9 Br	73 H_2_O, 4 NO_3_
R_cryst_/R_free_ f		0.198/0.205	0.184/0.217
r.m.s.d. bonds (Å)		0.015	0.021
r.m.s.d. angles (°)		1.7	1.8
Ramachandran plot (%) g		95.0/5.0/0.0/0.0	97.4/1.9/0.6/0.0

a Numbers in parentheses refer to the highest resolution shells.

b R_merge_  =  ∑_h_∑_i_ | I_i_(*h*) - <I(h)> |/∑_h_∑_i_I_i_(*h*), where I*_i_*(h) is the *i*th measurement of reflection *h* and <I(h)> is the weighted mean of all measurements of h.

c R_deriv_  =  ∑_h_||F_PH_ | - |F_P_||/∑_h_|F_P_|, where F_P_ and F_PH_ are the native and derivative structure factors, respectively.

d R_cullis_  =  ∑_h_|||F_PH_| - |F_P_|| - |F_H_||/∑_h_||F_PH_| - |F_P_|, where F_H_ is the calculated heavy atom structure factor.

e Phasing power is defined as (r.m.s F_H_/r.m.s lack-of closure).

f R  =  ∑_h_|F_obs_ - F_calc_|/∑_h_F_obs_, where F_obs_ and F_calc_ are the observed and calculated structure factor amplitudes, respectively. R_cryst_ and R_free_ were calculated using the working and test set, respectively.

g Residues in most favoured, additionally allowed, generously allowed and disallowed regions.

The Bim BH3 peptide binds into a surface groove formed by helices α2-5 of BHRF1 (Fig. 4A), in a similar manner to that previously observed for mammalian pro-survival Bcl-2 members such as Bcl-x_L_ [36] or the unrelated viral Bcl-2 protein M11L [27]. Bak BH3 binds in an equivalent manner (Fig. 4C), and the two complexes superimpose with an RMSD of only 0.6 Å over the entire BHRF1 backbone indicating their similarities. As the characteristic hydrophobic surface groove was absent in unliganded BHRF1 due to the close proximity of helices α3 and α4 (Fig. 4E; [35]), significant structural changes are required in order to accommodate Bim or Bak BH3 domains. These changes affect mainly α4, which is at a 120° angle to α3 in the BH3-bound form (Fig. 4A), compared to the near anti-parallel alignment in the free form (Fig. 4E). Overall, the free and bound BHRF1 structures (comparing Fig. 4A with 4B) superimpose with an RMSD of 3.5 Å, with most differences found within the BH3 binding groove. This is reminiscent of the movement observed in Bcl-x_L_, which in the ligand-free state displays a narrow binding groove [37]. However, upon ligand binding, both α3 and 4 helices move to widen the hydrophobic groove and allow binding [36]. In contrast, the movement of α3 that enables groove opening in BHRF1 upon ligand binding is much less pronounced.

The side-chain interactions contributing to the BHRF1-BH3 complexes are equivalent to those observed for mammalian pro-survival proteins such as Bcl-x_L_, with the four conserved hydrophobic BH3 residues of Bim (I58, L62, I65 and F69; numbering based on human Bim_L_) protruding into pockets within the BHRF1 hydrophobic binding groove (Fig. 4G). Similarly, Bak residues V74, L78, I81 and I85 (numbering based on human Bak) interact with the BHRF1 binding groove (Fig. 4H). In addition to the hydrophobic interactions, BHRF1 R100 forms a salt bridge with Bim D67 or with Bak D83 (Fig. 4G, 4H). This electrostatic interaction is also observed in complexes of Mcl-1 and Bcl-x_L_ with pro-apoptotic BH3 domains [36], [38] and even in a complex of Bcl-x_L_ with a peptide foldamer [39]. The conservation of aspartic acid in the mammalian BH3 ligands suggests that this interaction is of particular importance for complex formation, and indeed a BHRF1 R100A mutation reduces Bim binding and abolishes interaction with Bak [31].

### BHRF1 confers potent chemoresistance *in vivo*

**Figure 5:**
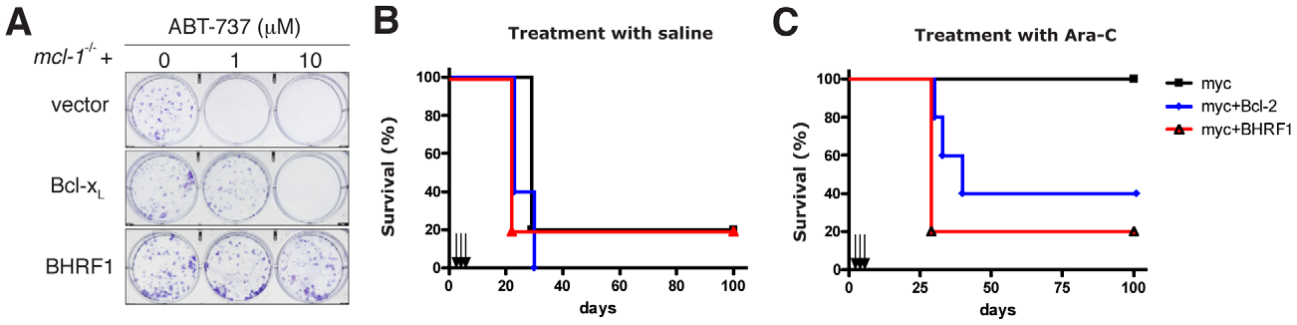
BHRF1 inhibits ABT-737 induced apoptosis and confers chemoresistance in a mouse model of Burkitt lymphoma. (A) Colony formation of *mcl*-1^−/−^ MEFs overexpressing BHRF1, Bcl-x_L_ or a control vector, 6 d after treatment with 0-10 µM ABT-737. (B, C) Kaplan-Meier survival plots of mice inoculated with Eµ-*myc* tumor cells overexpressing BHRF1, Bcl-2 (or an empty vector) and treated on d 4, 5 and 6 (arrows) afterwards with (B) saline or (C) 100 mg/kg cytosine arabinoside (Ara-C; given intra-peritoneally). N = 5 mice in each group. Expression of BHRF1 or Bcl-2 conferred chemoresistance (p = 0.014 and p = 0.049 respectively by log-rank test when compared to saline treated controls). However, there is no significant difference whether or not BHRF1 or Bcl-2 is expressed as a transgene (p = 0.85 by log-rank test).

Since BHRF1 engages BH3 domains using a hydrophobic groove (Fig. 4) in a manner equivalent to that of its mammalian counterparts, conserving key interactions, we asked whether ABT-737, a BH3 mimetic compound known to inhibit Bcl-2, Bcl-x_L_, and Bcl-w [40], [41], could also target BHRF1. In the absence of pro-survival Mcl-1, ABT-737 is a potent cytotoxic agent. However, cells expressing BHRF1 were completely insensitive to ABT-737, even at the highest dose tested (10 µM) (Fig. S2), and survived long-term (Fig. 5A). Similarly, recombinant BHRF1 did not bind ABT-737 in biosensor assays (IC_50_>20 µM, data not shown). As ABT-737 is ineffective and as BHRF1 can potently confer chemoresistance when tested in cultured cell lines (Fig. 1), we evaluated the impact of its expression in a transgenic mouse model of Burkitt lymphoma [11]. In the Eµ-*myc* mouse, *myc* is overexpressed in the B cell and drives an aggressive B cell leukemia/lymphoma syndrome that is very similar to human Burkitt lymphoma.

Malignant cells derived from sick Eµ-*myc* mice are readily transplantable into syngeneic wild-type recipients which succumb within four weeks to a disseminated disease if left untreated [42]. Treatment with Ara-C (Fig. 5C, black line), an agent used in the clinic for treating patients with Burkitt lymphoma, resulted in a sustained disease remission and survival with all mice alive and disease-free at 100 days with normal peripheral blood counts and spleen weights. In striking contrast, only a handful of treated mice that were inoculated with tumor cells overexpressing BHRF1 or Bcl-2 survive long-term (Fig. 5C). Comparable results were observed when two other cytotoxic agents, cyclophosphamide and etoposide, were used in similar efficacy studies (data not shown). Therefore BHRF1, like Bcl-2 [42], can potently confer chemoresistance in a mouse model of Burkitt lymphoma. Thus, it is highly likely that expression of BHRF1 will attenuate the response during treatment for EBV-driven malignancies.

### Concluding remarks

In this study, we have confirmed that EBV BHRF1 exerts its pro-survival function by directly inhibiting a sub-set of pro-apoptotic Bcl-2 family proteins Bid, Bim, Puma and Bak, presumably ones most critical for the virus. Our three-dimensional structures show that these interactions closely resemble those seen with mammalian pro-survival proteins such as Bcl-x_L_. In light of the importance of BHRF1 in certain Burkitt lymphomas [9], and its detection in other EBV-associated malignancies including nasopharyngeal carcinoma [43] and B cell lymphomas [44], the development of therapeutic inhibitors of BHRF1 may be highly desirable. The inability of current small molecule inhibitors of mammalian pro-survival proteins such as ABT-737 to inhibit BHRF1 will require novel small molecule antagonists to be developed. The successful development of small molecule inhibitors of mammalian pro-survival Bcl-2 family proteins [40] suggests that similar approaches might be applied to the development of BHRF1 inhibitors.

## Materials and Methods

### Recombinant proteins and binding experiments

BHRF1ΔC31 was cloned into pET DUET (Invitrogen) using *BamHI* and *EcoRI*, and expressed in *E.coli* BL21 DE3 pLysS. Cells were homogenized using an Avestin EmulsiFlex homogenizer in lysis buffer (50 mM Tris-HCl, pH 8.0, 150 mM NaCl, 10 mM BME). His-tagged BHRF1ΔC31 was purified using a nickel charged Hi-Trap chelating column (Amersham), eluted in lysis buffer with 250 mM imidazole and subjected to gel-filtration chromatography in 25 mM Hepes pH 7.5, 150 mM NaCl using a Superdex 200 column (GE Healthcare). Calorimetry data were collected on a VP-ITC (MicroCal) with BHRF1ΔC31 as previously described [45]. All peptides were purified by reverse-phase HPLC and dissolved as 2–5 mM stock solutions in water. The accession numbers for the peptides were: human Bim_L_ (AAC39594), human Puma (AAK39542), mouse Bmf (AAK38747), human Bad (NP_004313), human Bik (NP_001188), human Hrk (NP_003797), human Bid (NP_001187), human Noxa (NP_066950), human Bax (NP_620119), human Bak (NP_001179), mouse Mule (UniProt Accession code Q7TMY8; residues 1969–1994; PGGTTQEVGQLLQDMGDDVYQQYRSL) and mmBeclin (UniProt Accession code O88597, residue 103–128; DGGTMENLSRRLKVTGDLFDIMSGQT).

### Crystallization and structure determination

BHRF1 complexes with Bim or Bak BH3 were obtained by mixing BHRF1 with human Bim or Bak 26-mer peptide in a 1∶1.25 molar ratio and concentrating using a centricon (Millipore) to 10 mg/mL. Crystals were grown in sitting drops at 20°C in 1.2 M NaBr, 50 mM malic acid pH 4.0 (BHRF1:Bim) or 1.6 M NaNO_3_, 50 mM malic acid pH 4.4 (BHRF1:Bak). The crystals belong to space group P3_2_21 with a = b = 62.75 Å, c = 92.38 Å, α = β = 90°, γ = 120° (BHRF1:Bim) or a = b = 62.39 Å, c = 93.73 Å, α = β = 90°, γ = 120° (BHRF1:Bak). The asymmetric units contain 1 BHRF1:peptide complex. Diffraction data were collected from flash frozen crystals at 100 K at the Australian Synchrotron (beamline 3BM1) or at the Swiss Light Source (beamlines X06SA, X10SA) and processed with HKL2000. For the BHRF1:Bim complex, a heavy atom derivative was obtained by soaking crystals in mother liquor supplemented with 1 mM MeHgCl for 2 h. Hg sites were found and refined with Sharp. Clear and continuous electron density was obtained for residues 2–158 of BHRF1 and 51–72 of Bim. The final model was built with Coot [46], refined with Refmac5 [47] to a resolution of 1.5 Å and has a final R-factor of 0.198 (R-free 0.205). 95.0% of the residues are in the core regions of the Ramachandran plot, and no residues are in disallowed regions.

The BHRF1:Bak structure was solved by molecular replacement with PHASER [48] using the BHRF1:Bim structure as a search model. The final model was built with Coot and refined with Refmac5 to a resolution of 2.05 Å and has a final R-factor of 0.184 (R-free 0.217). 97.4% of the residues are in the core regions of the Ramachandran plot, and no residues are in disallowed regions. All data collection and refinement statistics are summarized in Table 1. The coordinates have been deposited in the Protein Data Bank (accession codes 2v6q, 2wh6, 2xpx). Figures were prepared using PyMol (DeLano Scientific).

### Mouse tumor model

Eµ-*myc* transgenic mice on an inbred C57BL/6 background with clinical evidence of lymphoma were culled by CO_2_ asphyxiation and lymphomatous tissue excised. Single-cell suspensions were obtained by manual sieving and stable Eµ*-myc* tumor cell cultures were established in FMA medium. Retroviral transduction using a pMSCV-IRES-GFP vector containing BHRF1 or Bcl-2 and sorting of GFP positive cells were conducted as previously described [11]. 1×10^6^ viable tumor cells in 100 µL PBS were injected intraperitoneally into syngeneic C57BL/6 mice. Ara-C or PBS was injected intraperitoneally in 100 µL total volume on days 4, 5 and 6. Mice were culled when sick (hindleg paralysis, tremor, lethargic, tumor nodule >1 cm diameter, >5% weight loss) and leukemia/lymphoma confirmed by the presence of peripheral blood leucocytosis and enlarged spleen and/or lymph nodes. Survival of cohorts of 5 mice was compared by log-rank test and Kaplan-Meier analysis using GraphPad Prism statistical software. All experiments were approved by an institutional ethics committee.

### Cell lines and tissue culture

MEF, HEK-293T and Phoenix packaging 293 cells were cultured in Dulbecco's Modified Eagles medium (DMEM) supplemented with 10% FCS. FDC-P1 cells were additionally supplemented with mouse IL-3 (1000 U/mL). Eµ-*myc* tumor cells were harvested from a symptomatic Eµ-*myc* transgenic mouse and cultured in FMA, a high glucose version of DMEM supplemented with 10% FCS, 50 µM 2-mercaptoethanol and 250 µM asparagine.

### Mammalian expression constructs

Epitope-tagged mammalian expression vectors for human Bcl-2 family proteins have been described previously [20], [49], [50], [51], [52]. All constructs were verified by sequencing. Details and constructs are available from the authors.

### Retrovirus production and transduction

To produce retroviral supernatant, 2.5×10^6^ ecotropic Phoenix packaging cells were seeded overnight in 10 cm tissue culture plates. Media was replaced with 5 ml serum-free DMEM containing 5 µg MSCV-based retroviral plasmid with 15 µL Lipofectamine (Invitrogen). After 24 h media was replaced with medium supplemented with 20% FCS and incubated for a further 24 h at 32°C. Viral supernatant was cleared of cell debris by centrifugation for 5 min at 1500 rpm. 500 µL filtered virus (0.45 µm, Millipore) was spin-infected onto target cells in a total volume of 1 mL media containing 4 µg/mL polybrene (Sigma) in 24 well plates at 32°C with 2500 rpm radial centrifugation for 45 min. Infection efficiency of MEFs were generally >90% and 20–30% for FDC-P1 cells.

### Cell survival assays and cytotoxic drugs

Cell death was induced by 0–100 µM etoposide (Pharmacia-Upjohn), 0–30 Gy γ-irradiation, 0–10 µM Ara C (Pharmacia-Upjohn), 0–100 nM staurosprorine (Sigma-Aldrich) or 0–10 µM ABT-737 (Abbott Laboratories). Cell viability was quantified by flow cytometric analysis of cells excluding 5 µg/mL propidium iodide (PI) (Sigma-Aldrich) using a FACScan (Becton Dickinson). Each time point was performed at least three times. For long-term colony assays using MEFs, cells were infected with GFP-expressing retroviral constructs, then treated with qVD.OPH (Enzyme Systems) to prevent cell death. After culture for 24 h, 200 GFP^+ve^ cells were sorted into 6-well plates. Colonies were stained and counted 6 d later.

### Immunofluorescence

Cells expressing mammalian FLAG-tagged pro-survival Bcl-2 proteins, BHRF1 or empty vector were washed in PBS, fixed in 1% paraformaldehyde/PBS (10 min, 4°C) and washed twice in KDS-BSS. Cells were incubated with 1∶1,000 primary anti-FLAG M2 (Sigma) antibody for 20 minutes, washed in KDS-BSS/0.02% saponin and then incubated with 1∶100 goat anti-mouse FITC or PE antibody (Southern Biotechnology) for 20 minutes before analysis on a FACScan (BD) using Cell Quest software (BD).

### Cytofluorometric determination of mitochondrial transmembrane potential and Bax/Bak activation

To assess mitochondrial transmembrane potential (Δψm), cells were incubated for 15 min at 37°C in buffer containing 40 nM 3,3′- dihexyloxacarbocyanine iodide (DiOC6[3]; Molecular Probes) before adding 10 µg/mL of PI. The cells were kept on ice until flow cytometric analysis. To assess the activation of Bax and Bak, HeLa cells were left untreated or pretreated with a proteasome inhibitor (10 µM MG-132; Calbiochem) or a wide-spectrum caspase inhibitor (100 µM zVAD.fmk; Bachem) for 1 h before treatment with 200 J/m^2^ UV-irradiation. Following UV irradiation, cells were fixed with 1% paraformaldehyde (5 min at room temperature) and then washed with buffer supplemented with 2% fetal bovine serum. Fixed cells were then incubated with the primary antibodies: 2 µg/mL anti-Bak Ab-1 (Calbiochem) or 5 µg/mL anti-Bax clone 3 (BD) diluted in FACS buffer supplemented with 0.3% saponin for 30 min on ice. Cells were then washed, before incubation with the appropriate secondary antibody, either FITC-conjugated goat-anti-mouse IgG (10 µg/ml; SouthernBiotech) to detect Bax activation or a biotin-conjugated anti-mouse (diluted 1∶200; SouthernBiotech) followed by Streptavidin-conjugated PE (diluted 1∶300; Caltag) to detect Bak activation. The samples were analyzed using a FACScan (BD).

### Subcellular fractionation

Fractionation of whole cell lysates into the soluble and pellet fractions has been previously described [54]. In brief, cells lysed in HMKEE buffer (20 mM Hepes, pH 7.2, 5 mM MgCl2, 10 mM KCl, 1 mM EDTA, 1 mM EGTA, and protease inhibitors) containing 250 mM sucrose and 0.025% digitonin (Calbiochem) were left on ice for 10 min, and then the organelles, cytoskeleton, and membranes were pelleted by centrifugation (13,000 rpm, 5 min at 4°C). The pellet was solubilized in RIPA buffer (150 mM NaCl, 1% Triton X-100, 0.5% deoxycholic acid, 0.1% SDS, 50 mM Tris-HCl, pH 8.0, and protease inhibitors). The protease inhibitors used include Pefabloc SC, soybean trypsin inhibitor, leupeptin, aprotinin, E64, and pepstatin (Sigma-Aldrich or Roche).

### Transient transfection, immunoprecipitation and immunoblotting

The transfection and metabolic labeling of HEK-293T cells with ^35^S-methionine/cysteine (NEN) as well as co-immunoprecipitation have been described [49], [50], [52]. Briefly, equivalent TCA-precipitable lysates were immunoprecipiated using the mouse monoclonal antibodies to FLAG (M2; Sigma), Glu-Glu (CRP) and control HA (HA.11; CRP) tags. The proteins were resolved by SDS:PAGE, transferred onto nitrocellulose membranes and detected by autoradiography after 20 h at −80°C. Immunoblotting was performed using mouse monoclonal antibodies to Bax (5B7; Sigma-Aldrich) cytochrome c (7H8.2C12; BD Pharmingen) and detected using HRP-conjugated secondary antibodies (Southern Biotechnology) revealed by enhanced chemiluminescence (ECL; Amersham Biosciences).

### Yeast colony assays

Yeast expression vectors were made by subcloning the cDNAs for full-length human Bcl-x_L_ and BHRF1, or human Bax and human Bak, respectively, into the pGALL(TRP1) and pGALS(LEU2) vectors [55]. *Saccharomyces cerevisiae* W303a cells were co-transformed with indicated plasmids and grown under selection. For the survival assays, the cells were spotted as 5-fold serial dilutions onto glucose (repressing, “OFF”) or galactose (inducing, “ON”) plates as previously described [28]. Plates were incubated for 48 h at 30°C and then photographed.

### Ethics statement

This study was carried out in strict accordance with the recommendations in the Guide for the Care and Use of Laboratory Animals of the National Health and Medical Research Council. The protocol was approved by the Committee on the Ethics of Animal Experiments of the Walter and Eliza Hall Institute of Medical Research (Permit Number: NKOT_07_008). All surgery was performed under sodium pentobarbital anesthesia, and all efforts were made to minimize suffering.

## Supporting Information

Figure S1 Pre-B-cell tumor cells derived from Eµ-*myc* transgenic mice were stably transfected with BHRF1, Bcl-2 or a control vector and exposed to etoposide (0–10 µM). Viability was assessed by PI staining after 24 h.
(0.17 MB TIF)

Figure S2 BHRF1 is not inhibited by ABT-737. Mcl-1 deficient MEF stably expressing BHRF1, Bcl x_L_ or a control vector were treated with ABT-737 (0–10 µM). Viability was assessed 8 h later by flow cytometry after propidium iodide staining.
(0.13 MB TIF)
