# Supplementary figures and images for: Structural Basis for Apoptosis Inhibition by Epstein-Barr Virus BHRF1
Source: PLoS Pathog. 2010 Dec 23;6(12):e1001236. doi: 10.1371/journal.ppat.1001236 (PMC3009601; doi:10.1371/journal.ppat.1001236)

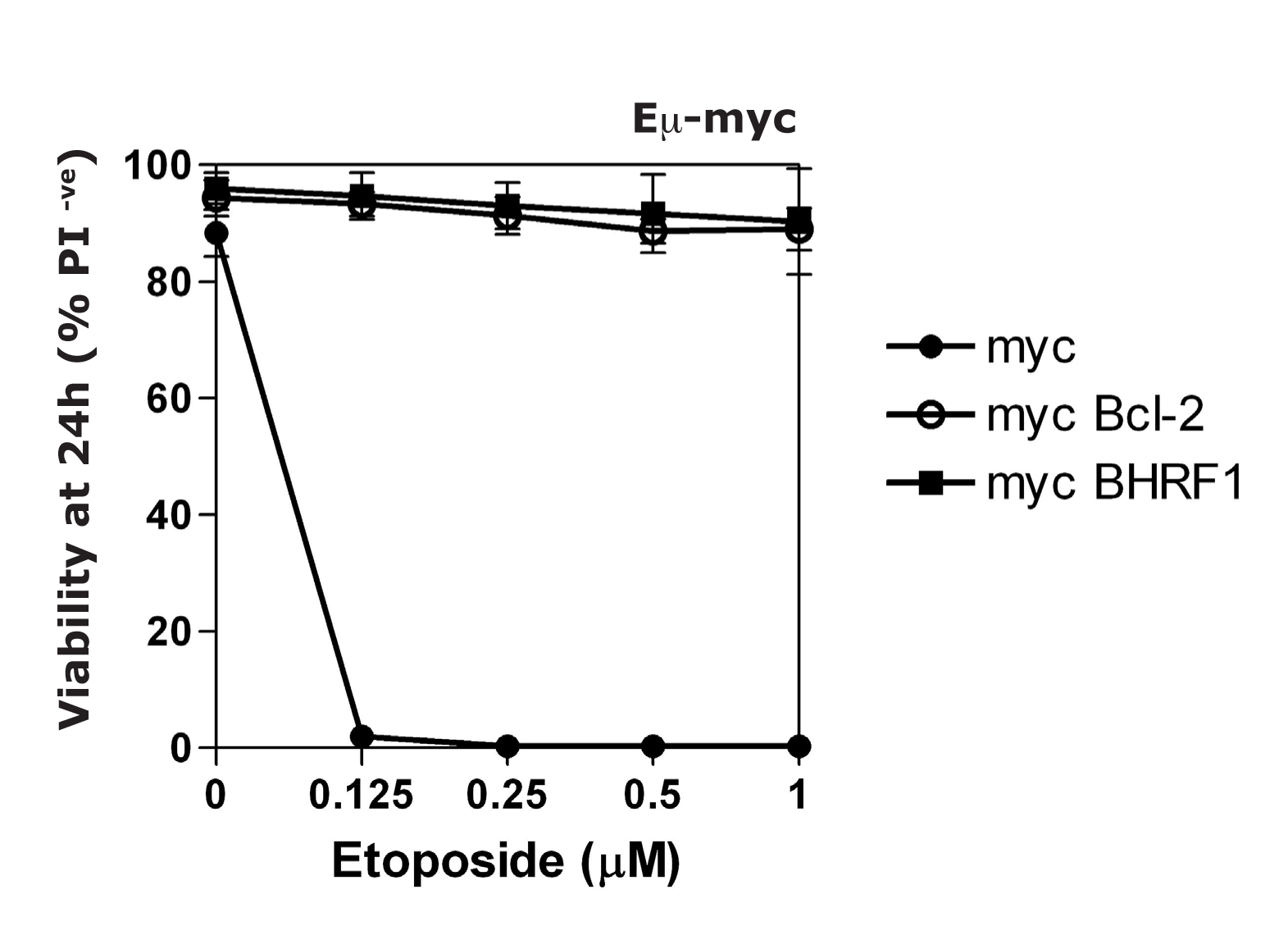

Supplement: Figure S1 — Pre-B-cell tumor cells derived from Eµ-myc transgenic mice were stably transfected with BHRF1, Bcl-2 or a control vector and exposed to etoposide (0–10 µM). Viability was assessed by PI staining after 24 h. (0.17 MB TIF) [file ppat.1001236.s001.tif]

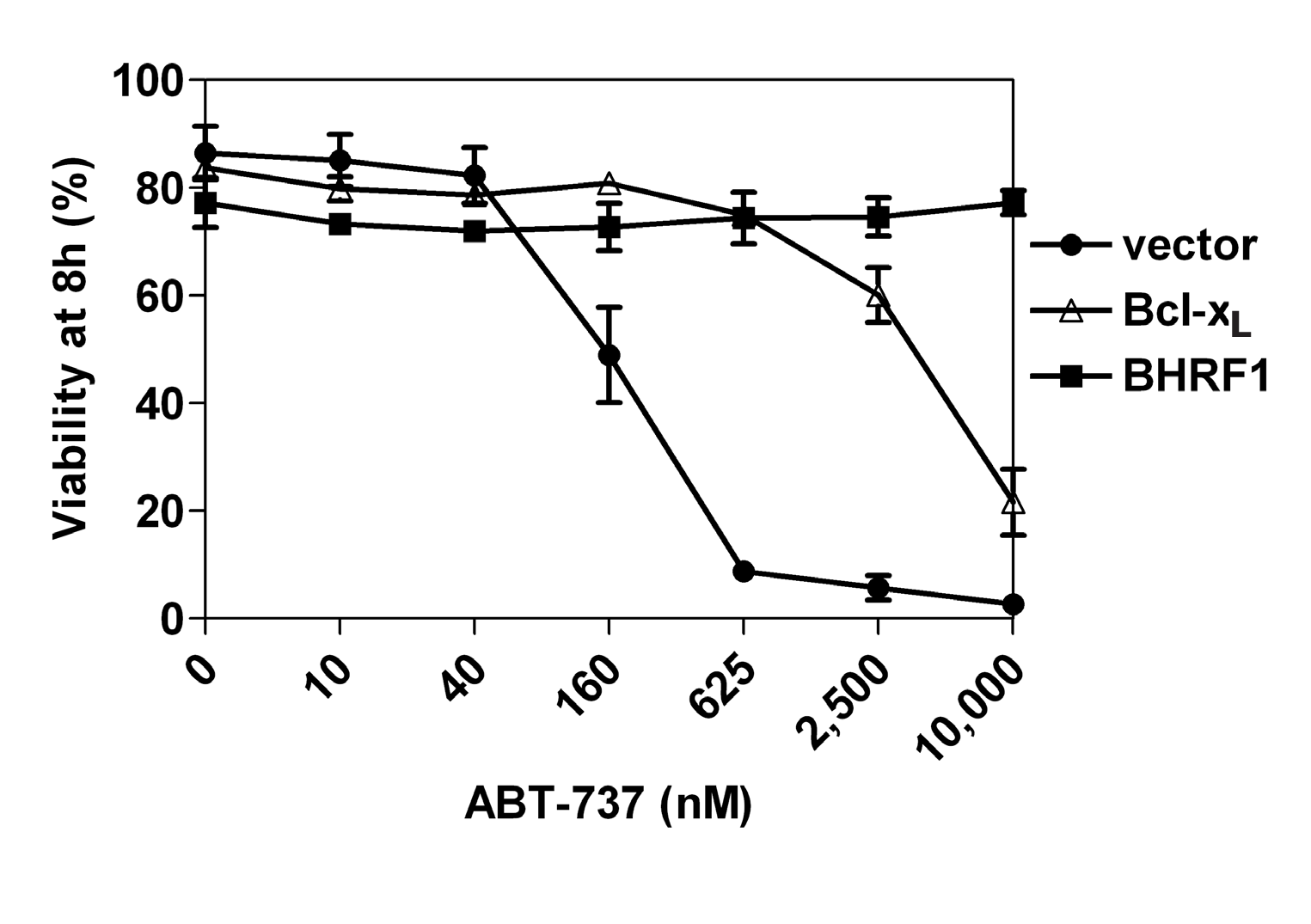

Supplement: Figure S2 — BHRF1 is not inhibited by ABT-737. Mcl-1 deficient MEF stably expressing BHRF1, Bcl xL or a control vector were treated with ABT-737 (0–10 µM). Viability was assessed 8 h later by flow cytometry after propidium iodide staining. (0.13 MB TIF) [file ppat.1001236.s002.tif]
